# Tumor cell PD-L1 expression is a strong predictor of unfavorable prognosis in immune checkpoint therapy-naive clear cell renal cell cancer

**DOI:** 10.1007/s11255-021-02841-7

**Published:** 2021-04-01

**Authors:** Katharina Möller, Christoph Fraune, Niclas C. Blessin, Maximilian Lennartz, Martina Kluth, Claudia Hube-Magg, Linnea Lindhorst, Roland Dahlem, Margit Fisch, Till Eichenauer, Silke Riechardt, Ronald Simon, Guido Sauter, Franziska Büscheck, Wolfgang Höppner, Cord Matthies, Ousman Doh, Till Krech, Andreas H. Marx, Henrik Zecha, Michael Rink, Stefan Steurer, Till S. Clauditz

**Affiliations:** 1 Institute of Pathology, University Medical Center Hamburg-Eppendorf, Martinistr. 52, 20246 Hamburg, Germany; 2 Department of Urology, University Medical Center Hamburg-Eppendorf, Hamburg, Germany; 3 Department of Urology, Itzehoe Medical Center, Itzehoe, Germany; 4 Department of Urology, Bundeswehr Hospital Hamburg, Hamburg, Germany; 5 Department of Urology, Regio Medical Center Elmshorn, Elmshorn, Germany; 6 Institute of Pathology, Clinical Center Osnabrueck, Osnabrück, Germany; 7 Department of Pathology, Academic Hospital Fuerth, Fuerth, Germany; 8 Department of Urology, Albertinen Clinic, Hamburg, Germany

**Keywords:** PD-L1, Renal cell carcinoma, Immune checkpoint therapy, Prognosis

## Abstract

**Background:**

PD-L1 expression predicts response to immune checkpoint inhibitors in renal cell carcinomas (RCC), but has also been suggested to be linked to poor patient outcome.

**Methods:**

We analyzed PD-L1 in > 1400 RCC in a tissue microarray format by immunohistochemistry. Results were compared with histological tumor type, parameters of cancer aggressiveness, and intratumoral CD8^+^ cytotoxic cells.

**Result:**

At a cut-off level of 5% PD-L1 positive tumor cells, PD-L1 positivity was seen in 6.3% of 633 clear cell RCC (ccRCC), 18.2% of 165 papillary RCC, 18.8% of 64 chromophobe RCC, and 41.7% of 103 oncocytomas. In ccRCC, PD-L1 positivity was significantly linked to high ISUP (*p* < 0.0001), Fuhrman (*p* < 0.0001), Thoenes grade (*p* < 0.0001), distant metastasis (*p* = 0.0042), short recurrence-free (*p* < 0.0001), and overall survival (*p* = 0.0002). Intratumoral CD8^+^ lymphocytes were more frequent in PD-L1 positive (1055 ± 109) than in PD-L1 negative ccRCC (407 ± 28; *p* < 0.0001). PD-L positive immune cells were seen in 8.2% of all RCC and 13.9% of papillary RCC. In ccRCC, PD-L1 positive immune cells were linked to high numbers of tumor-infiltrating CD8^+^ cells (*p* < 0.0001), high ISUP (*p* < 0.0001), Fuhrman (*p* = 0.0027), and Thoenes grade (*p* < 0.0001), and poor tumor-specific survival (*p* = 0.0280).

**Conclusions:**

These data suggest that PD-L1 expression in highly immunogenic RCCs facilitates immune evasion and contributes to cancer aggressiveness.

**Supplementary Information:**

The online version contains supplementary material available at 10.1007/s11255-021-02841-7.

## Introduction

Renal cell carcinoma (RCC) is one of the most common cancer types worldwide [1]. Localized tumors are generally treated by total or partial nephrectomy. For patients in need for a systemic therapy, several new drugs have recently gained approval and improved the prognosis of metastatic RCC [2, 3]. As in other cancer types, immune checkpoint inhibitors are in focus of current research [4–6]. In clear cell RCC, combinations of pembrolizumab (PD-1 inhibitor) and axitinib (VEGFR inhibitor), ipilimumab (CTLA-4 inhibitor) and nivolumab (PD-1 inhibitor), or avelumab (PD-L1 inhibitor) and axitinib showed superior survival as compared to standard therapies in phase III studies and are thus recommended and FDA approved as first-line systemic therapy in intermediate- and poor-risk patients [2, 7–10].

Clinical trials are currently investigating, whether adjuvant application of immune checkpoint inhibitors or other new drugs can improve the prognosis of kidney cancer patients at high risk for disease recurrence or progression after nephrectomy (Keynote-564, iMmotion010, Checkmate-914) [11]. If adjuvant treatment becomes standard of care, risk stratification will become more important than ever before, to enable optimal treatment decisions for individual patients. In this context, programmed cell death 1 ligand 1 (PD-L1) expression measurement is of particular interest. PD-L1 is one of the two programmed cell death 1 (PD-1) ligands and, thus, a part of an immune checkpoint system (PD-1/PD-L1) with widespread clinical application. PD-L1 expression—both on cancer cells and on tumor-infiltrating immune cells—predicts a favorable response to immune checkpoint inhibitors in various tumor types [12]. In RCC, several studies suggested that PD-L1 positivity is associated with a high number of tumor-infiltrating lymphocytes [13–20] and poor prognosis in cancers treated otherwise [15, 17, 19, 21–32]. A minority of studies came to different conclusions [20, 33–38]. The partially discrepant study results are likely to be caused by a lack of standardized procedures for PD-L1 measurement. Studies investigating PD-L1 expression by immunohistochemistry (IHC) have described positivity rates ranging from 5 to 57% for tumor cells [29, 39] and from 8 to 75% for immune cells [22, 40].

To generate more data on the potential prognostic role of PD-L1 expression in kidney cancer and its relationship with intratumoral lymphocytes, a cohort of 1476 RCC—all treated in the pre-immunotherapy era—was analyzed in a tissue microarray format (TMA) for PD-L1 expression on tumor cells and immune cells by IHC.

## Material and methods

### Patients

A set of TMAs was used containing one tissue core each from 1476 kidney tumors routinely diagnosed from nephrectomy specimen between 1994 and 2016 at the Institute of Pathology of the University Medical Center Hamburg-Eppendorf, Germany. All tumors had been reviewed according to the criteria described in the 2016 WHO classification by two pathologists with a special focus on genitourinary pathology (FB, CF) and ISUP (International Society of Urological Pathology) grading was performed for each tumor. Follow-up data were available for 531 of 808 clear cell RCC and 136 of 205 papillary RCC. Available study endpoint were overall survival, tumor-specific death, and recurrence-free survival, including patients without metastasis (M0) at the timepoint of surgery and patients with initial metastasis (M1) and additional progress after surgery. Density of CD8^+^ cells measured by IHC was available in 1315 cases from a previous study [41]. The TMA comprises three blocks, which had been used earlier [42]. The TMA manufacturing process was described in detail before [43]. In brief, from each donor tumor, one tissue core measuring 0.6 mm in diameter was taken from a tumor-containing tissue block. Clinical and pathological parameters of the arrayed tumors are summarized in Table 1. The mean follow-up time was 48 months. The use of archived remnants of diagnostic tissues for manufacturing of TMAs and their analysis for research purpose as well as patient data analysis has been approved by local laws (HmbKHG, §12) and by the local ethics committee (Ethics Commission Hamburg, WF-049/09). All work has been carried out in compliance with the Helsinki Declaration.

**Table 1:** Patient cohort

	Study cohort on TMA
	(*n* = 1476)
Follow-up	
Available (*n*)	848
Mean (months)	62
Median (months)	39
Age (years)	
< 50	202
50–70	384
70–90	729
Histology	
Clear cell RCC	808
Papillary RCC	205
Chromophobe RCC	81
Oncocytoma	127
UICC stage	
I	559
II	76
III	113
IV	102
pT category	
pT1	728
pT2	150
pT3–4	277
ISUP grade	
1	329
2	368
3	298
4	59
Fuhrman grade	
1	56
2	636
3	303
4	68
Thoenes grade	
1	376
2	594
3	93
pN category	
pN0	174
PN +	37
pM category	
pM0	175
pM +	97

Numbers do not always add up to 1476 in the different categories because of missing data

### Immunohistochemistry (IHC)

Freshly prepared TMA sections were immunostained on 1 day in one experiment. Slides were deparaffinized and exposed to heat-induced antigen retrieval for 5 min in an autoclave at 121 °C in pH9 Dako Target Retrieval Solution buffer (Agilent, CA, USA; #S2367). Primary antibody specific against PD-L1 protein (mouse monoclonal, MS Validated Antibodies, Hamburg, Germany, clone MSVA-011) was applied at 37 °C for 60 min at a dilution of 1:150. Bound antibody was then visualized using the EnVision Kit™ (Agilent, CA, USA; #K5007) according to the manufacturer’s instructions. Membranous PD-L1 staining of the kidney tumor cells and immune cells was separately interpreted. In tumor cells, different cut-offs based on the percentage of PD-L1 positive cells were used (≥ 1%, ≥ 5%, ≥ 10%, and ≥ 50%). In immune cells, PD-L1 staining were grouped in negative (no staining), few positive (few cells stained), and many positive (many cells stained). Density of CD8^+^ staining was evaluated in a previous study [41].

### PD-L1 antibody comparison

To evaluate the staining properties of the used anti-PD-L1 antibody MSVA-011 in comparison with the anti-PD-L1 antibody E1L3N (rabbit monoclonal, Cell Signaling, Danvers, Massachusetts; #13684)—which was most frequently used in earlier PD-L1 studies in kidney cancer—multiplex fluorescence IHC (mfIHC) was used. For mfIHC, the OPAL dye kit (Cat. # NEL811001KT, AKOYA Biosciences, Menlo Park, California, United States) was used. The experimental procedure was mainly performed according to the manufacturer’s instructions (AKOYA). Slides were initially boiled in an autoclave (30 min at 100–120 °C in pH9 buffer) for antigen retrieval. Antibodies to detect PD-L1 were stained sequentially and counterstained with diamidinoino-2-phenylindole (DAPI). One circle of antibody staining included peroxidase blocking, application of the first primary (MSVA-011) antibody, detection with a secondary HRP-conjugated antibody, fluorescence dye detection (Opal 570), and removal of the bound antibodies by microwave treatment (4 min at 100 °C and 5 min at a mean temperature of 93 °C). This cycle was repeated for the second primary (E1L3N, pH9, dilution 1:200) antibody and the second fluorescence dye (Opal 690). The same experiment was repeated with exchanged Opal dyes for both PD-L1 clones to ensure that the antibody comparison was independent from the used fluorochromes. Slides were mounted in antifade solution. Placenta and normal human tonsil samples were used as control tissue on every slide for the antibody comparison. Digital images of fluorescence stained slides were acquired with a Leica Aperio VERSA 8 automated epifluorescence microscope. Image analysis was performed using HALO™ software package (Indica Labs, USA) and included segmentation of individual cells (Supplementary Fig. 1A/B) to enable intensity measurements of PD-L1. The image analysis workflow has been described earlier [44]. To measure the co-expression of both anti-PD-L1 antibodies across 28 cancer microenvironments, the relationship between PD-L1 expression and density has been analyzed: correlation analysis of the PD-L1 expression level on individual cancer and immune cells revealed a high degree of co-expression (*r* = 0.929, *p* < 0.0001; Supplementary Fig. 1C). In addition, the number of PD-L1 positive inflammatory cells of both anti-PD-L1 clones was highly concordant in 28 representative cancer microenvironments (*r* = 0.941, *p* < 0.0001, Supplementary Fig. 1C).

### Statistics

Statistical calculations were performed with JPM 14 software (SAS Institute Inc, NC, USA) [45] and R version 3.6.1 (The R foundation) [46, 47]. The Pearson’s correlation coefficient was used to measure the relationship between PD-L1 intensities and densities. Contingency tables and the Chi-square test were performed to search for associations between PD-L1 and tumor subtypes and tumor phenotype. ANOVA test was used to determine associations between PD-L1 immunostaining and CD8^+ ^density. Survival curves were calculated according to Kaplan–Meier. The log-rank test was applied to detect significant survival differences between groups analysis of variance (ANOVA) tests were used to investigate the relationship between categorical and continuous data. A *p* value ≤ 0.05 was expected as statistically significant.

## Results

### Technical issue

PD-L1 expression on both tumor and immune cells was informative in 1036 (70.2%) of 1476 arrayed cancers in our IHC analysis. Reasons of non-informative cases (*n* = 440; 29.8%) included lack of tissue samples or absence of unequivocal tumor cells in the TMA spot.

### PD-L1 expression in kidney tumors

**Fig. 1:**
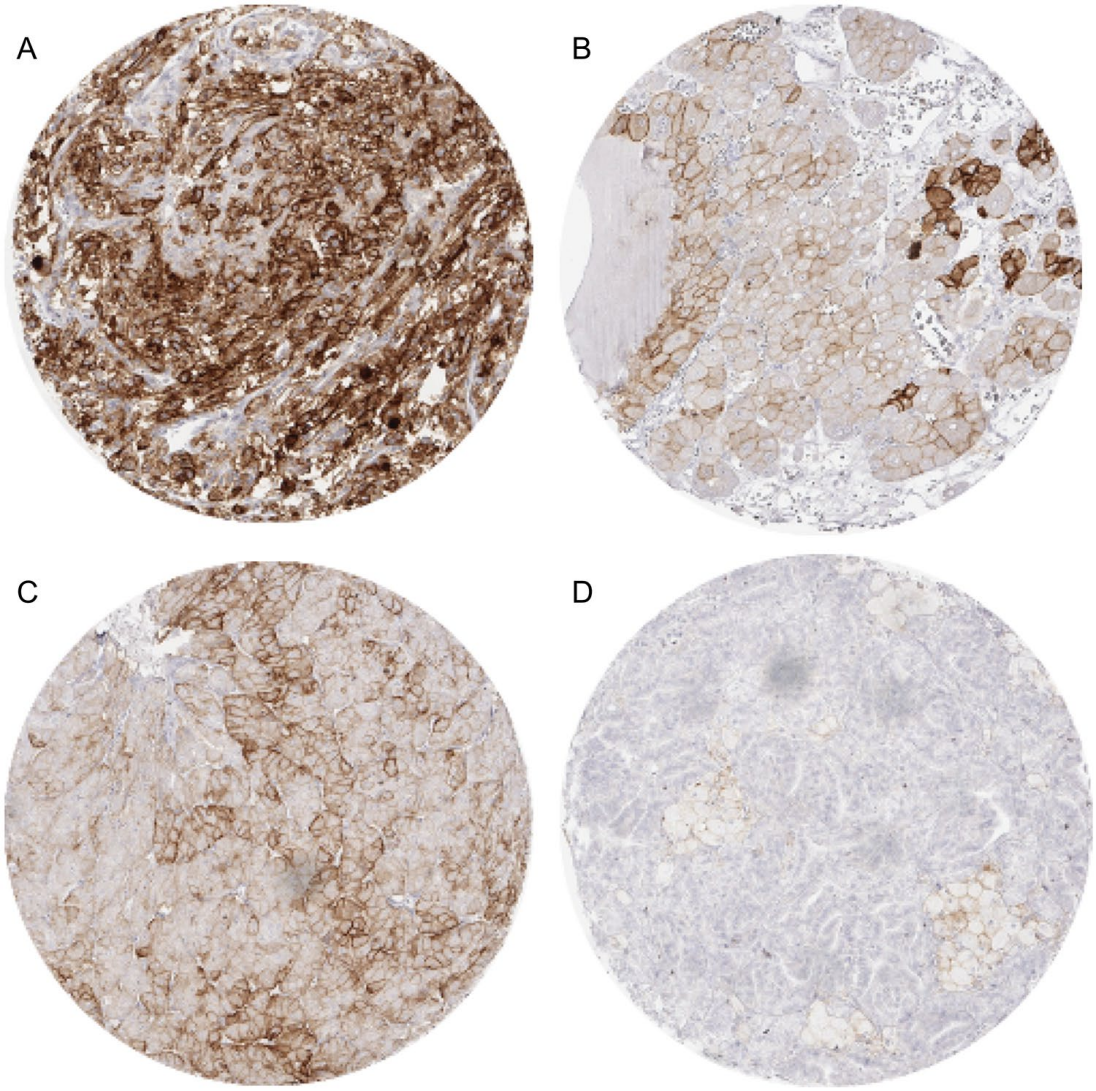
PD-L1 expression in renal cell tumors. PD-L1 immunostaining is diffuse and strong in a clear cell RCC (**a**), shows a mosaic pattern in an oncocytoma (**b**), and—with a higher rate of positive cells—in a chromophobe carcinoma (**c**). A papillary RCC without tumor cell staining shows abundant PD-L1 positivity in tumor-associated macrophages located in the cores of papillae (**d**)

In normal kidney, PD-L1 expression was not observed. In tumor cells and tumor-infiltrating immune cells, PD-L1 expression—if present—was membranous. The staining pattern generated by the antibodies MSVA-011 and E1L3N was largely identical (Supplementary Fig. 1). Using different cut-off levels to define positivity in tumor cells, PD-L1 positivity was detected at comparable frequencies of 12.9% (≥ 1% positive cells), 12.9% (≥ 5% positive cells), 11.0% (≥ 10% positive cells), and 4.9% (≥ 50% positive cells) in renal tumors. PD-L1 staining in tumor cells was often diffuse, sometimes focal within tumors and showed a mosaic pattern with a random appearing mixture of positive and negative cells. Representative images of PD-L1 positive tumors are given in Fig. 1. The frequency of PD-L1 expression varied between tumor subtypes. At a cut-off-level of 5% positive cells, PD-L1 expression in tumor cells was most commonly seen in oncocytomas (41.7%), clearly less frequent in chromophobe (18.8%) and papillary RCC (18.2%), and even less frequent in clear cell RCC (6.3%, Supplementary Table 1). In immune cells, PD-L1 expression was seen in 8.2% of 1036 tumors with highest frequencies in papillary RCCs (13.9%), followed by clear cell RCC (7.7%), and oncocytomas (5.8%, Supplementary Table 1).

### PD-L1 expression and tumor phenotype

**Fig. 2:**
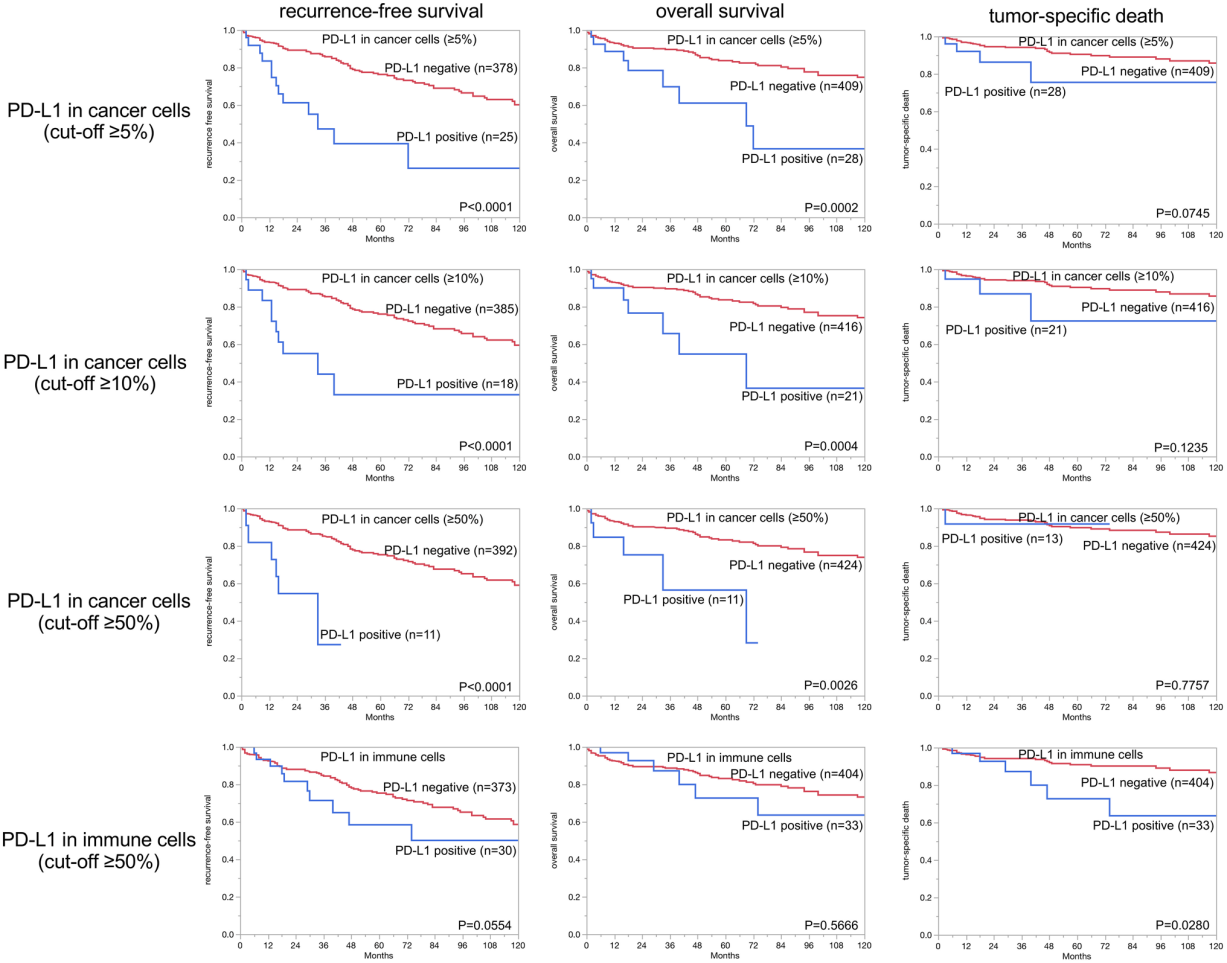
PD-L1 expression in cancer cells and immune cells and patient prognosis in clear cell RCCs

In clear cell RCC, PD-L1 expression in cancer cells and immune cells was related to adverse tumor features, including high ISUP (*p* ≤ 0.0010), high Fuhrmann (*p* ≤ 0.0030), and high Thoenes grade (*p* ≤ 0.0020) as well as short recurrence-free (*p* < 0.0001) and reduced overall survival (*p* ≤ 0.0030). This was largely regardless of the selected cut-off levels (Table 2, Fig. 2). In papillary RCCs, no association was found between PD-L1 expression and cancer phenotype or patient prognosis (Supplementary Fig. 2 and supplementary Table 2).

**Table 2:** PD-L1 expression in cancer cells and immune cells and tumor phenotype in clear cell RCC

	*n*	PD-L1 in cancer cells								PD-L1 in immune cells				
		PD-L1 positive cut-off 1%	*p* value	PD-L1 positive cut-off 5%	*p* value	PD-L1 positive cut-off 10%	*p* value	PD-L1 positive cut-off 50%	*p* value	None	Few	Medium	Many	*p* value
Clear cell cancers	633	6.3		6.3		5.1		2.4		92.3	4.7	2.5	0.5	
ISUP														
1	194	1	< 0.0001	1	< 0.0001	0.5	< 0.0001	0.5	0.0006	98.5	1	0	0.5	< 0.0001
2	206	3.4		3.4		2.9		0.5		93.7	2.4	3.9	0	
3	187	11.8		11.8		9.6		5.3		85.6	10.2	3.7	0.5	
4	38	21.1		21.1		18.4		7.9		86.8	7.9	2.6	2.6	
Fuhrmann														
1	32	3.1	< 0.0001	3.1	< 0.0001	0	< 0.0001	0	0.0003	100	0	0	0	0.0027
2	364	2.5		2.5		1.9		0.5		95.6	2.2	1.9	0.3	
3	190	11.1		11.1		8.9		4.2		85.8	10	3.7	0.5	
4	46	19.6		19.6		17.4		10.9		87	6.5	4.3	2.2	
Thoenes														
1	222	1.4	< 0.0001	1.4	< 0.0001	0.9	< 0.0001	0.5	0.0011	98.2	1.4	0.5	0	< 0.0001
2	349	6.9		6.9		5.7		2.3		90.3	6	3.4	0.3	
3	61	21.3		21.3		16.4		9.8		82	9.8	4.9	3.3	
UICC														
1	290	4.8	0.048	4.8	0.048	3.4	0.07	1.7	0.2324	93.8	4.5	1.4	0.3	0.0902
2	32	6.3		6.3		6.3		3.1		90.6	3.1	6.3	0	
3	84	9.5		9.5		7.1		6		88.1	4.8	7.1	0	
4	68	14.7		14.7		11.8		4.4		85.3	8.8	2.9	2.9	
Tumor stage														
pT1	371	4.9	0.0603	4.9	0.0603	4	0.2514	1.6	0.17	93.5	5.1	1.1	0.3	0.0796
pT2	67	4.5		4.5		4.5		1.5		95.5	1.5	3	0	
pT3–4	190	10		10		7.4		4.2		89.5	4.7	4.7	1.1	
Lymph node metastasis														
0	101	7.9	0.5109	7.9	0.5109	5	0.2529	2	0.355	92.1	5	3	0	0.1002
≥ 1	15	13.3		13.3		13.3		6.7		73.3	13.3	6.7	6.7	
Distant metastasis														
0	89	5.6	0.0042	5.6	0.0042	4.5	0.0132	2.3	0.0617	92.1	5.6	1.1	1.1	0.5741
≥ 1	68	20.6		20.6		16.2		8.8		85.3	10.3	2.9	1.5

### PD-L1 expression and density of CD8^+^ cells

Data on both PD-L1 expression in tumor cells or immune cells and CD8^+^ cell density were available for 633 clear cell RCC and 165 papillary RCC. Irrespective of the used cut-off levels, the intratumoral CD8^+^ density was significantly higher in clear cell RCCs with PD-L1 positive cancer cells than in PD-L1 negative clear cell RCCs. Clear cell RCCs with PD-L1 positive immune cells had also higher intratumoral CD8^+^ cell counts than clear cell RCCs without PD-L1 positive immune cells (*p* < 0.0001). In papillary RCCs, an association between PD-L1 expression in tumor cells and CD8^+^ density was generally not found (except cut-off level 50%). There was, however, a link between PD-L1 expression in immune cells and a high CD8^+^ density in papillary RCC (*p* = 0.0005, Table 3).

**Table 3:** PD-L1 expression in cancer cells and immune cells and density of CD8 positive cells

	Clear cell renal cell carcinomas			Papillary renal cell carcinomas		
	*n*	CD8 + density (cells/mm^2^)	*P*	*n*	CD8 + density (cells/mm^2^)	*p*
Tumor cells						
PD-L1 cut-off 1%						
Negative	593	407 ± 28.4	< 0.0001	135	219.9 ± 73.5	0.2539
Positive	40	1055.2 ± 109.3		30	317.9 ± 79.6	
PD-L1 cut-off 5%						
Negative	593	407 ± 28.4	< 0.0001	135	219.9 ± 73.5	0.2539
Positive	40	1055.2 ± 109.3		30	317.9 ± 79.6	
PD-L1 cut-off 10%						
Negative	601	411.8 ± 28.2	< 0.0001	137	216.8 ± 37.2	0.2320
Positive	32	1127.8 ± 122.3		28	325.3 ± 82.4	
PD-L1 cut-off 50%						
Negative	618	436.3 ± 28.4	0.0079	155	213.4 ± 34.5	0.0111
Positive	15	927.6 ± 182.1		10	573.2 ± 135.8	
Immune cells						
PD-L1						
Low (none + few)	614	412.3 ± 27.4	< 0.0001	142	188.4 ± 35.4	0.0005
High (medium + many)	19	1600.5 ± 155.9		23	524.1 ± 88.0	

## Discussion

In this study, PD-L1 immunostaining varied significantly between kidney cancer subtypes. This fits well to the well-known biological differences between different RCC subtypes (summarized in [48]). PD-L1 staining in tumor cells was significantly more frequent in papillary (18%) and chromophobe (19%), than in clear cell RCC (6%). Only few studies have earlier studied multiple RCC subtypes [18, 24, 26, 28, 49, 50] and the existing data on differences in PD-L1 expression between RCC subtypes are conflicting. Our observations are in line with studies that have also reported lower rates of PD-L1 positivity in clear cell compared to papillary (0–16% vs 27–32% [18, 26]) or in chromophobe RCC (0% vs 35% [18]). However, there are also studies showing equally high or even higher PD-L1 positivity rates in clear cell RCCs than in other renal tumor subtypes [26, 28, 49]. Of note, oncocytomas, the benign counterpart of chromophobe RCC showed the highest rate of PD-L1 positivity (42%) among the analyzed tumor subtypes. One earlier study had not found any PD-L1 expression in seven analyzed oncocytomas [18].

Clear cell RCC is the most common RCC subtype and has already been extensively analyzed for PD-L1 expression in earlier studies [14, 18–21, 25, 26, 28, 29, 31, 32, 35, 39, 40, 49, 51]. Our rate of 2–6% PD-L1 positive clear cell RCCs (depending on the cut-off level to define PD-L1 positivity) is in the lower range of published data with PD-L1 positivity ranging from 0 to 77% in studies analyzing 34–756 clear cell RCCs [14, 18–21, 25, 26, 28, 29, 31, 32, 35, 39, 40, 49, 51]. Reasons that are typically hold accountable for discrepant results in IHC studies include different antibodies, staining protocols, and criteria to define positivity. At least for RCC, the data do not suggest, that different binding properties of PD-L1 antibodies have led to the heterogeneous nature of existing data. 13 of the 33 earlier studies on PD-L1 in RCC have employed the antibody clone E1L3N, which shows highly similar staining properties as our antibody (Supplementary Fig. 1). These studies have described PD-L1 positivity to occur in 0–47% of clear cell RCC at a cut-off level of 1% or 5% stained cancer cells to define positivity [18, 19, 25, 28, 32, 35, 49, 51]. The quantity of tissue analyzed per patient and difficulties in the distinction of interspeared PD-L1 positive macrophages that are interspersed between cancer cells from true PD-L1 positive cancer cells might also contribute to the data diversity of PD-L1 immunohistochemistry in the literature. Especially, if studies are considered that utilize cut-off levels of 1% or 5% to define PD-L1 positivity, the positivity rate is significantly higher in 14 studies using large sections (11–77%; mean 28.9%) than in nine studies using TMAs (4–29%; mean 16.8%; *p* < 0.0001) [13–26, 28–40, 49–54]. While these data might suggest that relevant PD-L1 findings are missed on TMAs, it is also possible that interpretation errors—such as mistaking macrophages for tumor cells—are more likely to occur on large sections. The only large-scale study comparing IHC findings obtained from TMAs and corresponding large sections with clinical outcome data was on p53 immunostaining in breast cancer [55]. Torhorst et al. found 15–21% p53 positivity on each of four TMAs made from a cohort of 553 breast cancers but 43% positivity on large sections. As a link to patient survival was only seen for the p53 status obtained on the TMAs (*p* < 0.0001 each), but not for the large sections, it was concluded that prognostically irrelevant physiological or artificial p53 staining was overinterpreted on large sections but not on the TMAs.

PD-L1 positivity in tumor cells was strongly linked to unfavorable tumor phenotype and poor prognosis in this study. This was independent of the selected cut-offs and fits well with earlier data. 22 of 30 studies investigating the impact of PD-L1 expression on patient prognosis in 36–756 RCC patients have reported significantly worse outcomes in patients with PD-L1 positive tumors [13, 15, 19, 21–32, 39, 40, 49–51, 53]. Both the known functions of PD-L1 and the particularly frequent PD-L1 expression in oncocytoma—the only benign tumor included in our study—argue against a direct role of PD-L1 expression for tumor progression. Data from several laboratories have previously described that a high number of tumor-infiltrating lymphocytes are linked to poor patient prognosis in RCC [38, 41, 56, 57]. This is in sharp contrast to the majority of other cancer types, such as colorectal cancer, non-small cell lung cancer, breast cancer, and melanoma where a high number of tumor-infiltrating lymphocytes—considered as evidence for a strong anti-tumoral immune response—are strongly linked to favorable patient outcome [58–64]. It is tempting to speculate that the inverse prognostic impact of tumor-infiltrating lymphocytes in RCC is evidence for particularly efficient anti-immune mechanisms in these tumors protecting RCC cells from CD8^+^ cytotoxic T cells. The striking association between PD-L1 expression in cancer cells and a high number of intratumoral CD8^+^ cytotoxic T-lymphocytes may suggest that PD-L1 overexpression is driven by a lymphocyte-rich microenvironment and constitutes one of the mechanisms allowing for immune evasion and further progression of highly immunogenic RCCs.

Our rate of 8% of renal tumors with detectable PD-L1 positivity in tumor-associated immune cells is also in the lower range of the 6–75% reported in the literature [15, 22, 24, 26, 40, 51]. Whether PD-L1 expression is more relevant if it occurs in tumor cells or in immune cells is subject to intensive debate [65–67]. The significant but less striking prognostic role of PD-L1 in immune cells compared to tumor cells argues for a non-pivotal clinical role of PD-L1 expression on macrophages in RCCs that are not treated by immune checkpoint inhibitors. Given the significant association of PD-L1 positivity in immune cells with the number of tumor-infiltrating lymphocytes, it is possible that the amount of PD-L1 positive immune cells strongly depends on the amount of tumor-infiltrating lymphocytes in general, which is a well-known predictor of poor prognosis in RCC.

In summary, the data of this study demonstrate a strong link of PD-L1 expression in tumor cells with poor prognosis in RCC patients not treated with immune checkpoint inhibitors. The strikingly higher number of intratumoral CD8^+^ cytotoxic lymphocytes in PD-L1 positive compared to PD-L1 negative RCCs may suggest that PD-L1 overexpression is driven by a lymphocyte-rich microenvironment and reflects a pivotal component of the particularly efficient immune evasion mechanisms of RCCs.

## Supplementary Information

Below is the link to the electronic supplementary material.

Supplementary file1 (PDF 3803 KB)

## Data Availability

All data generated or analyzed during this study are included in this published article [and its supplementary information files].
