# Supplementary material for: Tumor cell PD-L1 expression is a strong predictor of unfavorable prognosis in immune checkpoint therapy-naive clear cell renal cell cancer
Source: Int Urol Nephrol. 2021 Apr 1;53(12):2493–503. doi: 10.1007/s11255-021-02841-7 (PMC8599390; doi:10.1007/s11255-021-02841-7)

Tumor cell PD-L1 expression is a strong predictor of unfavorable prognosis in immune checkpoint therapy-naive clear cell renal cell cancer

Katharina Möller[1], Christoph Fraune[1], Niclas C Blessin[1], Maximilian Lennartz[1], Martina Kluth[1], Claudia Hube-Magg[1], Linnea Lindhorst[1], Roland Dahlem[2], Margit Fisch[2], Till Eichenauer[2], Silke Riechardt[2], Ronald Simon[1#], Guido Sauter[1], Franziska Büscheck[1], Wolfgang Höppner[3], Cord Matthies[4], Ousman Doh[5], Till Krech[1,6], Andreas H Marx[1,7], Henrik Zecha[8], Michael Rink[2], Stefan Steurer[1], Till S Clauditz[1]

[1]Institute of Pathology, University Medical Center Hamburg-Eppendorf, Hamburg, German

[2]Department of Urology, University Medical Center Hamburg-Eppendorf, Hamburg, Germany

[3]Department of Urology, Itzehoe Medical Center, Itzehoe, Germany.

[4]Department of Urology, Bundeswehr Hospital Hamburg, Hamburg, Germany

[5]Department of Urology, Regio Medical Center Elmshorn, Elmshorn, Germany

[6]Institute of Pathology, Clinical Center Osnabrueck, Osnabrück, Germany

[7]Department of Pathology, Academic Hospital Fuerth, Fuerth, Germany

[8]Department of Urology, Albertinen Clinic, Hamburg, Germany

# Corresponding author: Dr. Ronald Simon, Institute of Pathology, University Medical Center Hamburg-Eppendorf, Martinistr. 52, 20246 Hamburg, Germany, Tel: +49 40 7410 57214, FAX +49 40 7410 55997, E-mail: R.Simon@uke.de

Supplementary Table 1. PD-L1 expression in tumor cells and immune cells and histotype

		PD-L1 in cancer cells								PD-L1 in immune cells				
Histotype	n	PD-L1 positive cut-off 1%	p vs clear cell cancer	PD-L1 positive cut-off 5%	p vs clear cell cancer	PD-L1 positive cut-off 10%	p vs clear cell cancer	PD-L1 positive cut-off 50%	p vs clear cell cancer	none	few	medium	many	p vs clear cell cancer
clear cell renal cell carcinoma	633	6.3		6.3		5.1		2.4		92.3	4.7	2.5	0.5	
papillary renal cell carcinoma	165	18.2	<0.0001	18.2	<0.0001	17	<0.0001	6.1	0.0252	86.1	8.5	3	2.4	0.0483
oncocytoma	103	41.7	<0.0001	41.7	<0.0001	33	<0.0001	13.6	<0.0001	94.2	3.9	1.9	0	0.7484
chromophobe renal cell carcinoma	64	18.8	0.0017	18.8	0.0017	17.2	0.001	9.4	0.0095	100	0	0	0	0.0203
clear cell (tubulo) papillary renal cell carcinoma	15	0		0		0		0		93.3	0	6.7	0	
nephroblastoma	17	5.9		5.9		5.9		0		82.4	17.6	0	0	
Xp11.2 translocation renal cell carcinoma	7	14.3		14.3		14.3		14.3		100	0	0	0	
collecting duct carcinoma	4	0		0		0		0		100	0	0	0	
metanephric adenoma	4	0		0		0		0		100	0	0	0	
tubulocystic renal cell carcinoma	2	0		0		0		0		0	0	0	0	
multilocular cystic clear cell renal cell neoplasm of low malignancy	2	0		0		0		0		100	0	0	0	
medullary carcinoma	1	0		0		0		0		100	0	0	0	
neuroendocrine carcinoma	1	0		0		0		0		100	0	0	0	
carcinoma no otherwise specified (NOS)	15	40		40		40		26.7		80	6.7	6.7	6.7	

Supplementary Table 2. PD-L1 expression in cancer cells and immune cells and phenotype of papillary RCC

			PD-L1 in cancer cells								PD-L1 in immune cells				
		**n**	**PD-L1 positive cut-off 1%**	**p value**	**PD-L1 positive cut-off 5%**	**p value**	**PD-L1 positive cut-off 10%**	**p value**	**PD-L1 positive cut-off 50%**	**p value**	**none**	**few**	**medium**	**many**	**p value**
	papillary cancers	165	18.2		18.2		17.0		6.1		86.1	8.5	3.0	2.4	
**ISUP**	1	35	14.3	0.4315	14.3	0.4315	14.3	0.5070	5.7	0.5987	88.6	8.6	2.9	0.0	0.9644
	2	76	15.8		15.8		14.5		4.0		85.5	7.9	4.0	2.6	
	3	51	25.5		25.5		23.5		9.8		84.3	9.8	2.0	3.9	
	4	1	0.0		0.0		0.0		0.0		100.0	0.0	0.0	0.0	
**Fuhrmann**	1	1	0.0	0.2087	0.0	0.2087	0.0	0.2503	0.0	0.1711	100.0	0.0	0.0	0.0	0.8111
	2	112	14.3		14.3		13.4		3.6		86.6	8.0	3.6	1.8	
	3	47	27.7		27.7		25.5		10.6		85.1	10.6	2.1	2.1	
	4	3	33.3		33.3		33.3		33.3		66.7	0.0	0.0	33.3	
**Thoenes**	1	44	13.6	0.6115	13.6	0.6115	13.6	0.7267	2.3	0.3620	88.6	6.8	2.3	2.3	0.6146
	2	110	20.0		20.0		18.2		7.3		84.6	10.0	3.6	1.8	
	3	9	22.2		22.2		22.2		11.1		88.9	0.0	0.0	11.1	
**UICC**	1	94	20.2	0.6597	20.2	0.6597	18.1	0.6206	5.3	0.2290	85.1	8.5	4.3	2.1	0.7294
	2	15	13.3		13.3		13.3		0.0		86.7	6.7	0.0	6.7	
	3	3	33.3		33.3		33.3		33.3		100.0	0.0	0.0	0.0	
	4	9	33.3		33.3		33.3		11.1		88.9	0.0	0.0	11.1	
**tumor stage**	pT1	117	18.8	0.4465	18.8	0.4465	17.1	0.4675	6.0	0.6808	87.2	7.7	3.4	1.7	0.8090
	pT2	32	12.5		12.5		12.5		3.1		81.3	9.4	3.1	6.3	
	pT3-4	10	30.0		30.0		30.0		10.0		90.0	10.0	0.0	0.0	

**lymph node metastasis**	0	14	14.3	0.4419	14.3	0.4419	14.3	0.4675	7.1	0.6087	85.7	7.1	0.0	7.1	0.5926
	≥1	7	28.6		28.6		28.6		14.3		85.7	0.0	0.0	14.3	
**distant metastasis**	0	25	12.0	0.0960	12.0	0.0960	8.0	0.0517	0.0	0.0404	84.0	8.0	0.0	8.0	0.5054
	≥1	4	50.0		50.0		50.0		25.0		75.0	0.0	0.0	25.0	

Supplementary Figure 1: Multiplex fluorescence IHC of the used PD-L1 antibodies MSVA-011 (green) and E1L3N (red) revealed a high degree of co-expression (orange) (A and B). The PD-L1 expression and the density of PD-L1 positive cells was highly concordant across 28 cancer microenvironments (C).

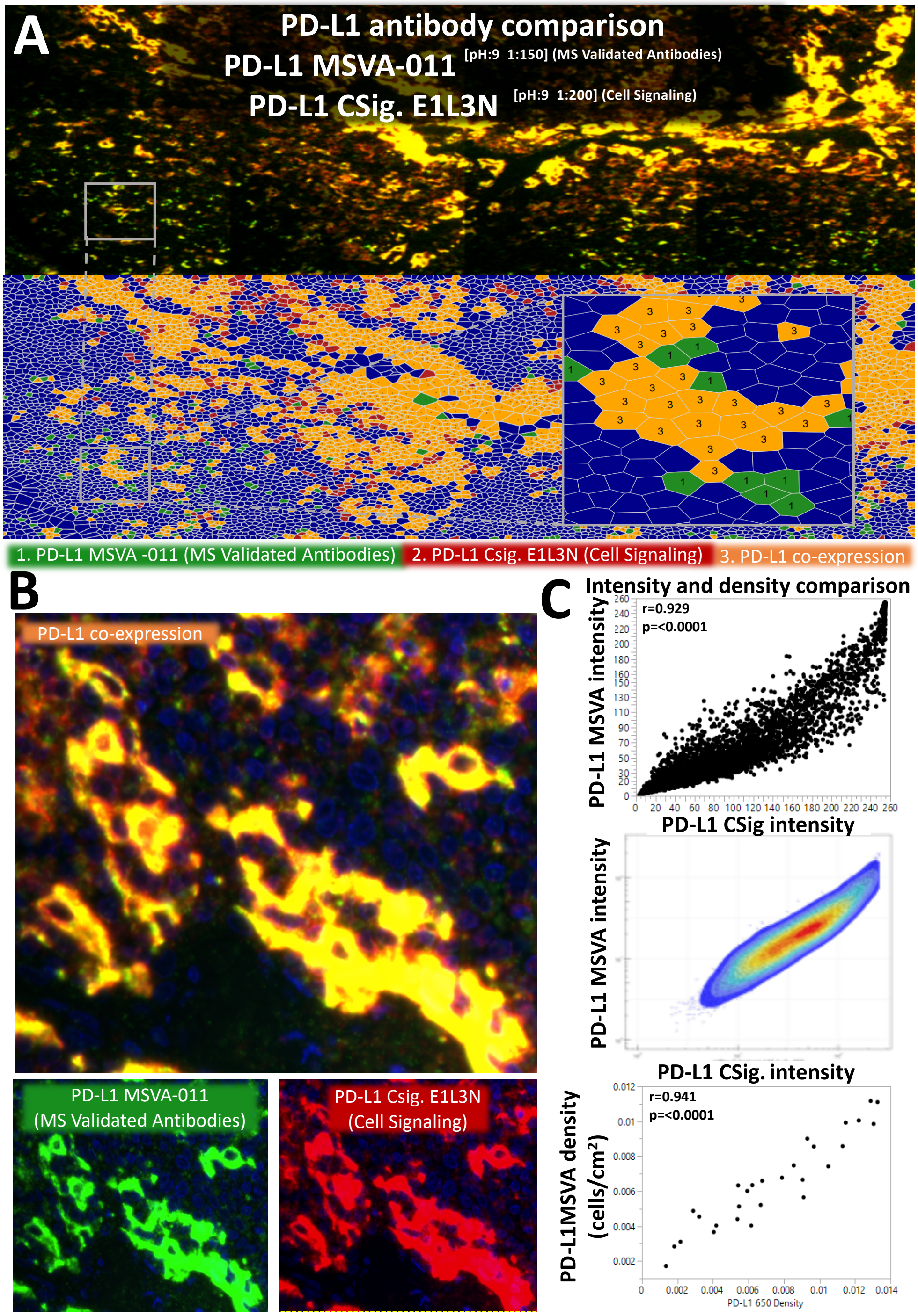

Supplementary Figure 2. PD-L1 expression in cancer cells and immune cells and patient prognosis in papillary RCCs

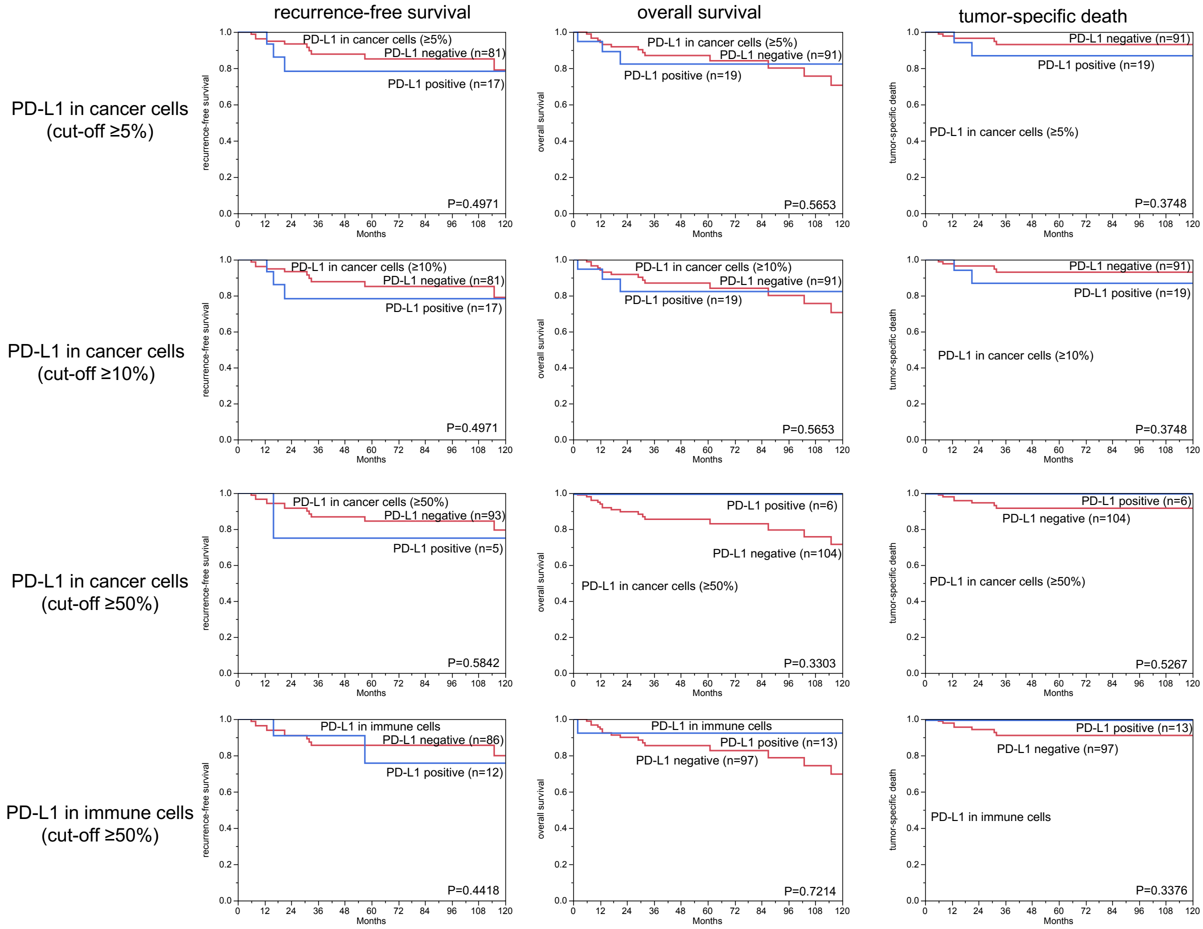

Supplement: Supplementary file 1 — Supplementary file1 (PDF 3803 KB) [file 11255_2021_2841_MOESM1_ESM.pdf]
